# Multi-locus sequence analysis unveils a novel genus of filarial nematodes associated with ticks in French Guiana[Fn FN1]

**DOI:** 10.1051/parasite/2024015

**Published:** 2024-03-15

**Authors:** Marjorie Bruley, Olivier Duron

**Affiliations:** MIVEGEC, University of Montpellier (UM), Centre National de la Recherche Scientifique (CNRS), Institut pour la Recherche de la Développement (IRD) Avenue Agropolis 34090 Montpellier France

**Keywords:** Filarial nematodes, Dipetalonema, tick, French Guiana

## Abstract

Filarial nematodes of the *Dipetalonema* lineage include tick-borne filarioids that infect both domestic and wild vertebrate hosts, but they remain understudied in many cases. In this study, we conducted a molecular characterization of a *Dipetalonema*-like filarioid (DLF) recently identified in two tick species in French Guiana, South America. While the *cox1* mitochondrial gene was the sole marker initially sequenced for describing DLF, its classification and phylogenetic relationship with other members of the *Dipetalonema* lineage were unclear. Therefore, we better characterized DLF through the sequencing of six additional gene markers and conducted phylogenetic analyses. Based on this multi-locus typing scheme, DLF exhibited significant divergence from known genera and species of filarioids, or other sequences available in public databases, suggesting its potential classification as a novel genus within the *Dipetalonema* lineage. Phylogenetic analyses further unveiled a close evolutionary relationship between DLF and all other filarioids associated with Acari (ticks and mites) within a robust monophyletic subclade in the *Dipetalonema* lineage. Overall, these findings confirm the existence of a specialized, Acari-borne group of filarioids and underscore the need for comprehensive investigations into their epidemiology and potential impact on animal health.

## Abstract (fr)

Les filaires de la lignée *Dipetalonema* comprennent des espèces transmises par les tiques qui infectent à la fois des hôtes vertébrés domestiques et sauvages, mais qui restent sous-étudiées dans de nombreux cas. Dans cette étude, nous avons réalisé une caractérisation moléculaire d’un filarioïde ressemblant à *Dipetalonema* (FRD) récemment identifié dans deux espèces de tiques en Guyane française. Alors que la séquence du gène mitochondrial *cox1* était le seul marqueur génétique initialement séquencé pour décrire FRD, sa classification et sa relation phylogénétique avec d’autres membres de la lignée *Dipetalonema* étaient incertaines. Par conséquent, nous avons caractérisé plus précisément DLF en séquençant six gènes supplémentaires et en réalisant des analyses phylogénétiques. Sur la base de ce typage multi-locus, FRD présentait une divergence significative par rapport aux genres et espèces connus de filarioïdes, ou à d’autres séquences disponibles dans les bases de données publiques, suggérant sa classification potentielle en tant que nouveau genre au sein de la lignée *Dipetalonema*. Les analyses phylogénétiques ont en outre révélé une relation évolutive étroite entre FRD et tous les autres filarioïdes associés aux tiques et acariens au sein d’un sous-clade monophylétique dans la lignée *Dipetalonema*. Dans l’ensemble, ces résultats confirment l’existence d’un groupe spécialisé de filarioïdes transmis par les tiques et acariens et soulignent la nécessité d’études approfondies sur leur épidémiologie et leur impact potentiel sur la santé animale.

## Introduction

Ticks are vectors of major viruses, bacteria, and protozoan parasites of medical and veterinary significance [24, 33, 34]. However, surveys of tick-borne pathogens often neglect filarial nematodes from the family Onchocercidae, commonly referred to as filariae or filarioids, while these parasites are regularly detected in most tick genera [1, 4, 6, 10, 11, 17, 29, 36, 39, 43, 45, 46, 50, 51]. Microscopic observations and molecular typing consistently categorize most tick-associated filarioids into the genera *Acanthocheilonema*, *Monanema*, *Yatesia*, and *Cercopithifilaria* [1, 4, 6, 10, 11, 17, 29, 3929, 35 36, , 43, 45, 46, 50, 51], although most were initially classified in the genus *Dipetalonema* [7, 9, 23]. Phylogenetic analyses based on molecular and morphological data further showed that the genera *Acanthocheilonema*, *Monanema*, *Yatesia*, and *Cercopithifilaria* (all associated with ticks), as well as *Cruorifilaria* (not yet associated with a vector, but detected in ticks [17]), *Litomosoides* (associated with parasitic mites) and *Dipetalonema* (associated with biting midges), cluster in a monophyletic clade of filarioids, termed the *Dipetalonema* lineage or the ONC4 clade, within the family Onchocercidae [7, 9, 23, 36].

Experimental infection assays and field observations have confirmed tick vector competence for filarioids of the genera *Acanthocheilonema*, *Monanema*, *Yatesia*, and *Cercopithifilaria*. Ticks feeding on infected vertebrates ingest microfilariae, which can develop up to the viable infective stage in a few weeks and are further excreted with saliva during biting, establishing specific tick-borne infection cycles [3–5, 8, 10, 13–15, 20, 32, 38, 40, 41, 44, 49, 52, 53]. This vector competence extends to major tick genera, including *Ixodes*, *Rhipicephalus*, *Amblyomma*, *Haemaphysalis*, *Hyalomma*, and *Ornithodoros*, further emphasizing the effectiveness of ticks as vectors for filarioids of the *Dipetalonema* lineage [3–5, 8, 10, 13–15, 20, 32, 38, 40, 41, 44, 49, 52, 53]. These filarioids also survive transstadially in ticks since the development from microfilariae to infective larvae occurs only while the tick is off-host, that is, during ecdysis from tick larva to nymph or from nymph to adult [41, 49].

In a recent survey of ticks in French Guiana, South America, molecular analysis and phylogenetic studies revealed the presence of novel filarioids belonging to the *Dipetalonema* lineage in several tick species [17]. Based on cytochrome c oxidase subunit I (*cox1*) mitochondrial gene sequences, all but one of these filarioids are distinct to already known species of *Dipetalonema* lineage. Indeed, in the Cayenne tick *Amblyomma cajennense* (Fabricius, 1787) and in the opossum tick *Ixodes luciae* Sénevet, 1940, one filarioid, provisionally named *Dipetalonema*-like (DLF hereafter), showed a *cox1* gene sequence substantially divergent from other species and genera of the *Dipetalonema* lineage [17]. DLF could be of health concern since it was detected in *A. cajennense* [17], the predominant tick species biting humans in South America [16]. This feature may not apply to *I. luciae*, as it is a specialized tick species with a primary feeding preference for opossums [16]. However, while DLF has been detected in 6% of field specimens of *A. cajennense* [17], no further data are currently available on this filarioid.

In this study, we conducted an extended molecular characterization of DLF previously detected in *A. cajennense* and *I. luciae* in French Guiana. The *cox1* gene sequence was the only genetic marker used for its description [17], but this marker exhibits limited resolution for inferring the evolutionary history in the family Onchocercidae [36]. Using infected field specimens of *A. cajennense* and *I. luciae*, we thus characterized DLF through the sequencing of six additional genes (*MyoHC*, *hsp70*, *rbp1*, 12S rRNA, 28S rRNA, and 18S rRNA) previously used for inferring the Onchocercidae phylogeny [36]. We further examined their genetic proximity with other filarioid species, including all known members of the *Dipetalonema* lineage, under a phylogenetic framework.

## Materials and methods

### Tick collection

A collection of 10 DNA templates from *A. cajennense* (*n* = 8) and *I. luciae* (*n* = 2) infected by DLF was used for the present analysis. All templates were obtained from field specimens collected on vegetation through flagging (questing ticks) or on opossums (engorged ticks) in French Guiana in 2016 and 2017 (Table 1). Each DNA template was obtained from individual extraction of tick whole body using a DNeasy Blood and Tissue Kit (QIAGEN, Hilden, Germany), following manufacturer instructions. For each DNA template, infection by DLF had previously been confirmed through *cox1* gene sequencing [17]. Use of the genetic resources was approved by the French Ministry of the Environment under reference #TREL19028117S/156, in compliance with the Access and Benefit Sharing procedure implemented by the *Loi pour la Reconquête de la Biodiversité*.

**Table 1:** List and origin of DLF-infected tick specimens examined in this study.

Host species	Sampling location, date	Stage, sex	Feeding status
Cayenne tick, *Amblyomma cajennense*	Kourou, 2016	1 nymph, 1 adult (female)	Questing ticks
	Matoury, 2016	6 nymphs	Questing ticks
Opossum tick, *Ixodes luciae*	Cayenne, 2017	2 adults (1 male, 1 female)	Engorged ticks (collected on opossum)

### Multi-locus typing of the *Dipetalonema*-like filarioid

Fragments of six genes (*MyoHC*, *hsp70*, *rbp1*, 12S rRNA, 28S rRNA, and 18S rRNA) were amplified using simple, semi-nested or nested PCR assays adapted from Lefoulon *et al.* [36]. Gene features, primers and PCR conditions are detailed in Table S1. Simple PCR amplifications were performed in a total volume of 25 μL containing ca. 20 ng of genomic DNA, 8 mM of each dNTP (Thermo Scientific, Waltham, MA, USA), 10 mM of MgCl_2_ (Thermo Scientific), 7.5 μM of each of the internal primers, 2.5 μL of 10×PCR buffer (Thermo Scientific), and 1.25 U of Taq DNA polymerase (Thermo Scientific). Nested and semi-nested PCR amplifications were performed as follows: the first PCR run with the external primers was performed in a 10 μL volume containing ca. 20 ng of genomic DNA, 3 mM of each dNTP (Thermo Scientific), 8 mM of MgCl_2_ (Roche Diagnostics), 3 μM of each primer, 1 μL of 10 × PCR buffer (Roche Diagnostics), and 0.5 U of Taq DNA polymerase (Roche Diagnostics). A 1 μL aliquot of the PCR product from the first reaction was then used as a template for the second round of amplification. The second PCR was performed in a total volume of 25 μL and contained 8 mM of each dNTP (Thermo Scientific), 10 mM of MgCl_2_ (ThermoScientific), 7.5 μM of each of the internal primers, 2.5 μL of 10 × PCR buffer (Thermo Scientific), and 1.25 U of Taq DNA polymerase (Thermo Scientific).

All PCR amplifications were performed under the following conditions: initial denaturation at 94 °C for 3 min, cycles of denaturation (35–40 cycles, depending on gene fragment size) (94 °C, 30 s), annealing (Tm = 50–55 °C, depending on primers, 30 s), extension (72 °C, 1 min–1 min 30 s, depending on gene fragment size), and a final extension at 72 °C for 5 min (Table S1). To prevent possible contamination, first and second PCR runs were physically separated from one another, in entirely separate rooms. Negative (water) controls were included in each PCR assay. All PCR products were visualized through electrophoresis in a 1.5% agarose gel. All amplicons were purified and sequenced in both directions (EUROFINS, Luxembourg). Sequence chromatograms were cleaned with Chromas Lite (http://www.technelysium.com.au/chromas_lite.html), and alignments were performed using ClustalW, implemented in the MEGA software package (https://www.megasoftware.net/). New sequences obtained in this study were deposited in GenBank under accession numbers PP182382–PP182391 (*MyoHC*), PP182371–PP182380 (*hsp70*), PP182391–PP182401 (*rbp1*), PP196371–PP196380 (12S rRNA), PP196417–PP196426 (28S rRNA), and PP196384–PP196393 (18s rRNA).

### Molecular phylogenetic analyses

Phylogenetic analyses were based on sequence alignments of the filarioid *MyoHC*, *hsp70*, *rbp1*, 12S rRNA, 28S rRNA, and 18S rRNA gene sequences obtained in this study. Analyses also included the filarioid *cox1* gene sequences (GenBank accession numbers OR030080–OR030087, OR030094, and OR030095) previously obtained from the same *A. cajennense* and *I. luciae* specimens by Binetruy and Duron [17]. Sequences of other filarioids obtained from GenBank, including representative members of the *Dipetalonema* lineage (*Acanthocheilonema*, *Yatesia*, *Cercopithifilaria*, *Cruorifilaria*, *Litomosoides*, and *Dipetalonema*) and of other filarial nematodes were also included in the phylogenetic analyses (Table S2). The Basic Local Alignment Search Tool (BLAST; https://blast.ncbi.nlm.nih.gov/blast/Blast.cgi) was used to find additional sequences available on GenBank. The Gblocks program with default parameters was used to obtain non-ambiguous sequence alignments [22]. Tree-based phylogenetic analyses were performed using maximum-likelihood (ML) analyses using the MEGA software package (https://www.megasoftware.net/). The evolutionary models that best fit the sequence data were determined using the Akaike information criterion. Clade robustness was assessed by bootstrap analysis using 1,000 replicates. We further conducted a phylogenetic network analysis based on uncorrected P distances using the Neighbor-net algorithm [21] implemented in SPLITSTREE [30]. The resulting phylogenetic networks generalize the trees by allowing cross-connections between branches, which might display conflicting signals in the phylogenetic data set [21].

## Results

### Multi-locus typing of the *Dipetalonema*-like filarioid

The DLF *MyoHC*, *hsp70*, *rbp1*, 12S rRNA, 28S rRNA, and 18S rRNA gene sequences were amplified from the 10 DNA templates (*A. cajennense*, *n* = 8; *I. luciae*, *n* = 2). All sequences were easily readable without double peaks, indicating a confident degree of primer specificity for filarioid PCR amplifications. On the basis of DNA sequencing, we characterized only one allele for each of the six genes. The DLF *MyoHC*, *hsp70*, *rbp1*, 28S rRNA, and 18S rRNA gene sequences were distinct from sequences available in public databases, and showed 83.2–98.9% pairwise nucleotide identities (depending on gene sequence) with other members of the *Dipetalonema* lineage, including *Acanthocheilonema*, *Monanema*, *Yatesia*, and *Cercopithifilaria* spp. (Table 2). Comparisons with filarioids other than members of the *Dipetalonema* lineage showed lower pairwise nucleotide identities for these gene sequences although the 12S rRNA sequences exhibited the highest pairwise nucleotide identities with filarioids of uncertain phylogenomic position (Table 2).

**Table 2:** Best nucleotide identities of DLF *MyoHC*, *hsp70*, *rbp*, 12S rRNA, 28S rRNA, and 18S rRNA gene sequences obtained in this study with sequences available in GenBank.

Gene	Best matches in public databases (% nucleotide identity, GenBank accession number)
*MyoHC*	*Acanthocheilonema viteae* (91.77%, KP760213); *Acanthocheilonema odendhali* (91.07%, KP760212)
*hsp70*	*Monanema martini* (85.01%, KP760443); *Acanthocheilonema viteae* (84.71%, KP760411)
*rbp1*	*Dipetalonema robini* (92.57%, KP760280); *Dipetalonema caudispina* (92.37%, KP760274)
12S rRNA	*Micipsella iberica* (83.18%, MW928503); Onchocercidae sp. GK-2015 (82.52%, KR676614)
28S rRNA	*Dipetalonema gracile* (97.45%, KP760371); *Dipetalonema robini* (97.09%, KP760374)
18S rRNA	*Yatesia hydrochoerus* (98.94%, KP760166); *Cercopithifilaria rugosicauda* (98.94%, KP760124)

### Phylogeny of the *Dipetalonema*-like filarioid

**Figure 1:**
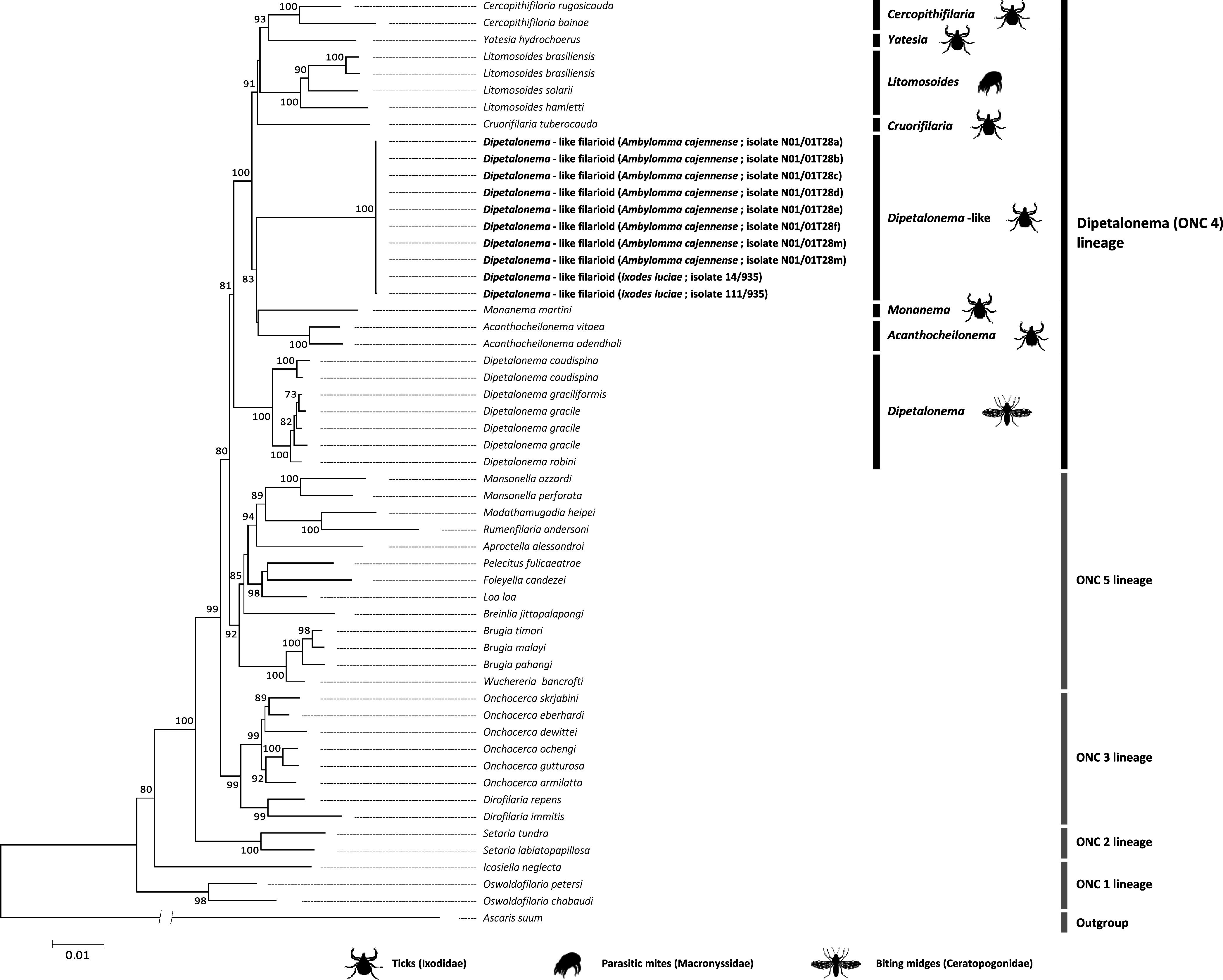
Phylogeny of onchocercid filarioids constructed using maximum-likelihood (ML) estimations based on concatenated *MyoHC*, *hsp70*, *rbp1*, 12S rRNA, 28S rRNA, 18S rRNA, and *cox1* nucleotide sequences with a total of 2,935 unambiguously aligned bp (best-fit approximation for the evolutionary model: GTR+G+I). Major genera of the *Dipetalonema* lineage (*Acanthocheilonema*, *Yatesia*, *Cercopithifilaria*, *Cruorifilaria*, *Monanema*, *Litomosoides*, and *Dipetalonema*), including representative species with indication of vector range (ticks, biting midges, parasitic mites), are indicated. Numbers at nodes indicate percentage support of 1,000 bootstrap replicates. Only bootstrap values >70% are shown. The scale bar is in units of substitution/site.

**Figure 2:**
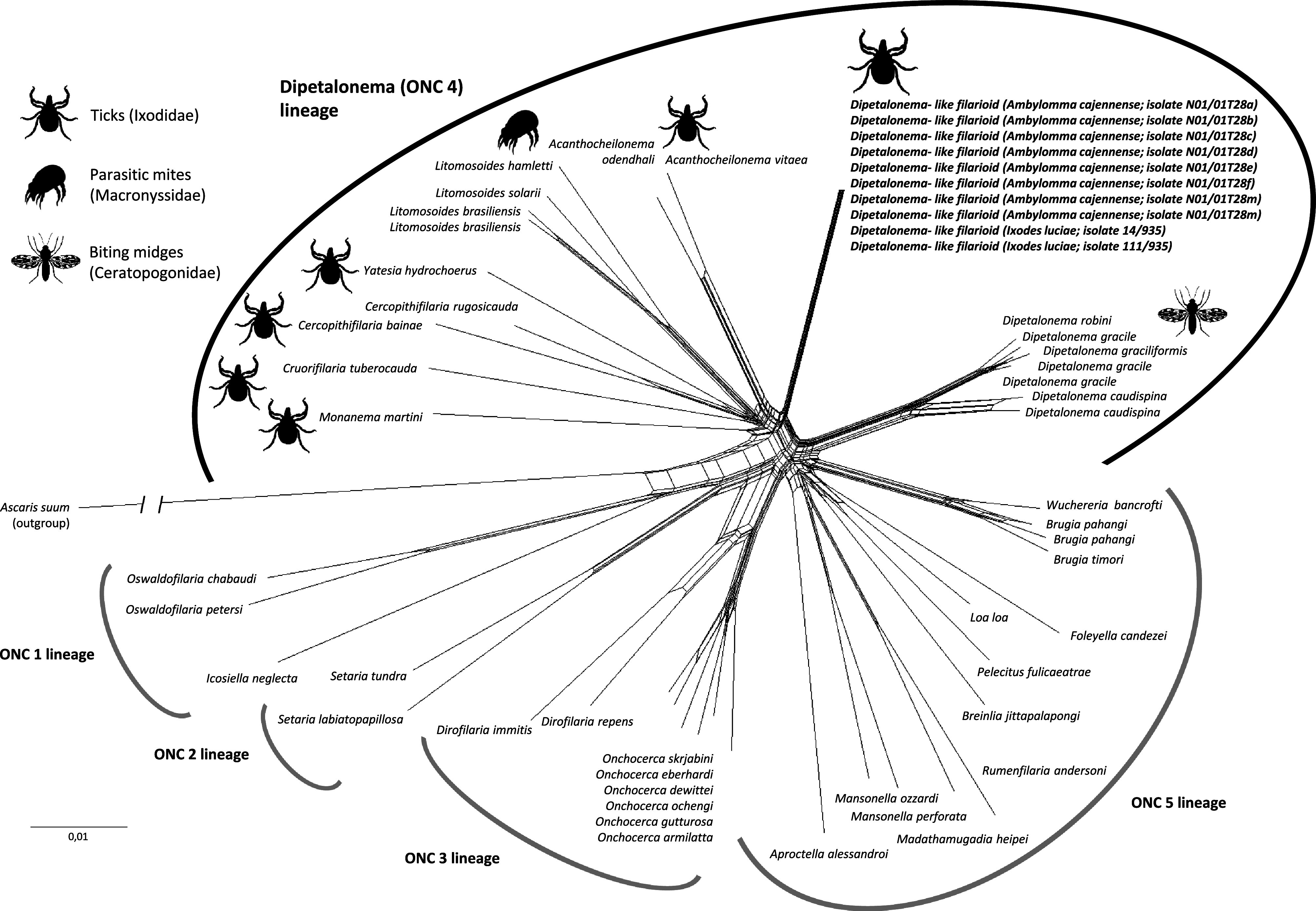
Phylogenetic network of onchocercid filarioids based on concatenated *MyoHC*, *hsp70*, *rbp1*, 12S rRNA, 28S rRNA, 18S rRNA, and *cox1* nucleotide sequences with a total of 2,935 unambiguously aligned bp. Major genera of the *Dipetalonema* lineage (*Acanthocheilonema*, *Yatesia*, *Cercopithifilaria*, *Cruorifilaria*, *Monanema*, *Litomosoides*, and *Dipetalonema*), including representative species with indication of vector range (ticks, biting midges, parasitic mites), are indicated. The scale bar is in units of substitution/site.

ML and phylogenetic network analyses based on *MyoHC* (717 bp), *hsp70* (561 bp), *rbp1* (500 bp), 12S rRNA (470 bp), 28S rRNA (436 bp), 18S rRNA (660 bp), and *cox1* (649 bp) nucleotide sequences were further conducted to examine the phylogenetic proximity of DLF with other filarioids. For any given gene, ML estimations gave similar tree topologies with minor differences, but also harbored some polytomies due to insufficient phylogenetic information. We thus conducted analyses using the 4, 083 bp (2, 935 unambiguously aligned bp) concatenated *MyoHC*, *hsp70*, *rbp1*, 12S rRNA, 28S rRNA, 18S rRNA, and *cox1* gene set (Figs. 1 and 2).

The ML and network analyses based on the concatenated dataset produced congruent phylogenetic trees with no major differences (Figs. 1 and 2). All phylogenetic reconstructions revealed a clustering of DLF with the genera *Cercopithifilaria*, *Cruorifilaria*, *Litomosoides*, *Yatesia*, *Acanthocheilonema*, *Monanema*, and *Dipetalonema* in a single monophyletic clade, the *Dipetalonema* lineage, distinct from other members of the family Onchocercidae. Phylogenetic reconstructions further revealed the division of the *Dipetalonema* lineage into two monophyletic subclades supported by high bootstrap values (Figs. 1 and 2):

1. The first subclade comprised DLF and members of the genera *Acanthocheilonema*, *Monanema*, *Cercopithifilaria*, *Yatesia*, *Cruorifilaria*, and *Litomosoides*. Within this subclade, DLF formed a branch substantially divergent from all other genera, but was more related to *Acanthocheilonema* species. Remarkably, all these filarioids are naturally associated with Acari, either with ticks (for DLF, *Acanthocheilonema*, *Monanema*, *Cercopithifilaria*, *Yatesia*, and *Cruorifilaria*), or with parasitic mites (for *Litomosoides*).
2. The second subclade comprised only *Dipetalonema* spp., which are filarioids specifically associated with biting midges.

As a result, the phylogenetic partitioning of the *Dipetalonema* lineage into two monophyletic subclades correlates with specialization for distinct types of arthropod vectors, Acari *vs.* dipterans.

## Discussion

In this study, we show that DLF displays substantial differences in its gene sequences compared to known genera and species within the family Onchocercidae, as well as any other sequences available in public databases. This observation lends support to the hypothesis that it could be a novel genus of filarioid with the *Dipetalonema* lineage. Furthermore, phylogenetic analyses unveil a close evolutionary relationship between DLF and all other filarioids associated with Acari (ticks and mites): these filarioids cluster together in a robust monophyletic subclade within the *Dipetalonema* lineage. These findings, consistent with earlier observations by Lefoulon et al. [36], suggest the presence of a monophyletic group of filarioids that has evolved a specialization for Acari as specific vectors.

Analyses of DNA gene sequence similarities and phylogenetics both confirm that DLF is divergent from other members of the *Dipetalonema* lineage. No morphological data are currently available for DLF but it may share morphological similarities with other members of the *Dipetalonema* lineage. Adults of these species have a long tail, a buccal capsule divided into two (or three) segments, more or less atrophied for specialized species, and a caudal extremity with two subterminal lappets [7, 23, 32]. Interestingly, opossums could be vertebrate hosts of DLF since *I. luciae* is a specialized tick species that feeds primarily on opossums [16]. Under this assumption, DLF may have previously been observed in opossums: previous studies have identified four filarioid species, all showing typical morphological features of the *Dipetalonema* lineage, *i.e*., *Acanthocheilonema pricei*, *Cercopithifilaria didelphis*, *Skrjabinofilaria skrjabini*, and *Cherylia guyanensis*, in South American opossums [8, 27]. However, only morphological data, and no molecular data, are currently available for these four filarioid species, which prevents us from concluding whether one of these already described species is a DLF.

The clustering of all Acari-associated filarioids in a monophyletic subclade, separate from those transmitted by blood-feeding dipterans, strengthens the conclusion that ticks serve as specific vectors for certain filarioids. It also implies that these filarioids are well adapted to tick physiology, life-cycle and behavior. Earlier experimental assays have confirmed that ticks are competent vectors of filarioids of the *Dipetalonema* lineage [3, 5, 20, 33, 39, 40, 43, 48, 51, 52]. These observations include the filarioid *Cherylia guianensis*, primarily isolated from a gray and black four-eyed opossum, which can normally develop in *Ixodes* ticks [8]. Furthermore, the detection of DLF from questing (unfed) *A. cajennense* ticks that have already digested their previous blood meals, have further moulted, and are seeking vertebrates for their next blood meal suggests that *A. cajennense* can acquire and stably maintain infection through transstadial transmission [17], as also observed for other members of the *Dipetalonema* lineage [40, 48]. For animals, the risk of acquiring a DLF infection is currently unknown, but surveys of dogs and capybaras infected by other filarioids of the *Dipetalonema* lineage revealed skin issues, chronic polyarthritis, anemia, and kidney and pulmonary damage [18, 19, 25, 26, 28, 41].

In conclusion, ticks transmit a broader range of infectious agents than any other arthropod vector, but their role as vectors of filarioids is less well-documented. The recurring identification of the *Dipetalonema* lineage species in major tick genera on most continents [2, 12, 17, 19, 31, 35, 37, 42, 46, 47] confirms that these are widespread but overlooked tick-borne parasites. Further research is needed to understand their pathogenicity, epidemiology, developmental cycles, and transmission mechanisms by ticks, including DLF in *A. cajennense* and *I. luciae*.

## Supplementary material

The Supplementary materials of this article are available at https://www.parasite-journal.org/10.1051/parasite/2024015/olm. *Table S1*: List and description of primers used in this study for DLF molecular typing (adapted from Lefoulon et al., 2015). *Table S2*: List of GenBank accession numbers for *MyoHC*, *hsp70*, *rbp*, 12S rRNA, 28S rRNA, and 18S rRNA gene sequences used in phylogenetic analyses.

## References

[1] Aeschlimann A, Burgdorfer W, Matile H, Peter O, Wyler R. 1979. Aspects nouveaux du rôle de vecteur joué par *Ixodes ricinus* L. en Suisse. Note préliminaire. Acta Tropica, 36, 181–191. 41427

[2] Almeida GLG, Vicente JJ. 1984. *Cercopithifilaria bainae* sp. n. parasita de *Canis familiaris* (L.) (Nematoda, Filarioidea). Atas da Sociedade de Biologia do Rio de Janeiro, 24, 18.

[3] Anteson RK. 1968. Biological studies of *Monanema marmotae* (Webster 1967) a filarioid parasite of the woodchuck *Marmota monax canadensis*. University of Connecticut: Storrs, Connecticut.

[4] Bain O. 1972. Recherches sur la morphogénèse des Filaires chez l’hôte intermédiaire. Annales de Parasitologie Humaine et Comparée, 47, 251–303.

[5] Bain O. 1967. Biologie larvaire et mécanisme de transmission de la Filaire *Dipetalonema viteae*. Annales de Parasitologie Humaine et Comparée, 42, 211–267. 5624529

[6] Bain O, Aeschlimann A, Chatelanat P. 1982. Présence, chez des tiques de la région de Genève, de larves infestantes qui pourraient se rapporter à la filaire de chien *Dipetalonema grassii*. Annales de Parasitologie Humaine et Comparée, 57, 643–646. 6891996

[7] Bain O, Baker M, Chabaud AG. 1982. Nouvelles données sur la lignée *Dipetalonema* (Filarioidea, Nematoda). Annales de Parasitologie Humaine et Comparée, 57, 593–620. 6891995

[8] Bain O, Petit G, Jacquet-viallet P, Houin R. 1985. *Cherylla guyanensis* n. gen., n. sp., – Filaire d’un Marsupial sud-américain, transmise par tique. Annales de Parasitologie Humaine et Comparée, 60, 727–737.

[9] Bain O, Casiraghi M, Martin C, Uni S. 2008. The nematoda filarioidea: Critical analysis linking molecular and traditional approaches. Parasite, 15, 342–348. 18814705 10.1051/parasite/2008153342

[10] Baltazard M, Chabaud AG, Minou A. 1952. Cycle évolutif d’une filaire parasite de mérion. Comptes Rendus Hebdomadaires des Séances de l’Académie des Sciences, 234, 2115–2117. 12979339

[11] Beaver PC, Burgdorfer W. 1984. A microfilaria of exceptional size from the ixodid tick, *Ixodes dammini*, from Shelter Island, New York. Journal of Parasitology, 70, 963–966. 6527192

[12] Bezerra-Santos MA, de Macedo LO, Nguyen VL, Manoj RR, Laidoudi Y, Latrofa MS, Beugnet F, Otranto D. 2022. *Cercopithifilaria* spp. in ticks of companion animals from Asia: new putative hosts and vectors. Ticks and Tick-Borne Diseases, 13, 101957. 35504199 10.1016/j.ttbdis.2022.101957

[13] Bianco AE. 1984. Laboratory maintenance of *Monanema globulosa*, a rodent filaria with skin-dwelling microfilariae. Zeitschrift für Parasitenkunde, 70, 255–264.

[14] Bianco AE, Muller RL. 1977. A hard tick as vector of a new rodent filaria. Transactions of the Royal Society of Tropical Medicine and Hygiene, 71, 383.

[15] Bianco AE, Nelson GS, Muller R. 1983. Biology of *Monanema globulosa*, a rodent filaria with skin-dwelling microfilariae. Journal of Helminthology, 57, 259–278.

[16] Binetruy F, Chevillon C, de Thoisy B, Garnier S, Duron O. 2019. Survey of ticks in French Guiana. Ticks and Tick-Borne Diseases, 10, 77–85. 30224310 10.1016/j.ttbdis.2018.09.003

[17] Binetruy F, Duron O. 2023. Molecular detection of *Cercopithifilaria*, *Cruorifilaria* and *Dipetalonema*-like filarial nematodes in ticks of French Guiana. Parasite, 30, 24. 37404115 10.1051/parasite/2023027 PMC10321233

[18] Bolio ME, Montes AM, Gutierrez C, Alonso FD, Bernal LJ, Sauri C, Rodríguez Vivas RI. 2002. Hallazgos clínicos en perros parasitados por *Dipetalonema dracunculoides*. Archivos de Medicina Veterinaria, XXXIV, 283–286.

[19] Boyd M, Santoro D, Craft WF, Ginn PE, Childress AL, Wellehan JFX, Walden HS. 2019. Dermatitis caused by autochthonous *Cercopithifilaria bainae* from a dog in Florida, USA: clinical, histological and parasitological diagnosis and treatment. Veterinary Dermatology, 30, 68–e20. 30474318 10.1111/vde.12701

[20] Brianti E, Otranto D, Dantas-Torres F, Weigl S, Latrofa MS, Gaglio G, Napoli E, Brucato G, Cauquil L, Giannetto S, Bain O. 2012. *Rhipicephalus sanguineus* (Ixodida, Ixodidae) as intermediate host of a canine neglected filarial species with dermal microfilariae. Veterinary Parasitology, 183, 330–337. 21831524 10.1016/j.vetpar.2011.07.031

[21] Bryant D, Moulton V. 2004. Neighbor-net: an agglomerative method for the construction of phylogenetic networks. Molecular Biology and Evolution, 21, 255–265. 14660700 10.1093/molbev/msh018

[22] Castresana J. 2000. Selection of conserved blocks from multiple alignments for their use in phylogenetic analysis. Molecular Biology and Evolution, 17, 540–552. 10742046 10.1093/oxfordjournals.molbev.a026334

[23] Chabaud AG, Bain O. 1976. La lignée *Dipetalonema*: Nouvel essai de classification. Annales de Parasitologie Humaine et Comparée, 51, 365–397. 988773

[24] Dantas-Torres F, Chomel BB, Otranto D. 2012. Ticks and tick-borne diseases: A One Health perspective. Trends in Parasitology, 28, 437–446. 22902521 10.1016/j.pt.2012.07.003

[25] Eberhard ML, Morales GA, Orihel TC. 1976. *Cruorifilaria tuberocauda* gen. et sp. n. (Nematoda: Filarioidea) from the Capybara, *Hydrochoerus hydrochaeris* in Colombia. Journal of Parasitology, 62, 604–607. 957038

[26] Espinosa N, Rosero A, Villegas CL, Garcia IC, Gaviria-Cantin T, Nieto AP, Ferro BE, Nieto Ramirez LM. 2022. First report of *Acanthocheilonema reconditum* outbreak in canines with clinical signs of anemia from Southwestern Colombia. Pathogens, 11. 10.3390/pathogens11121434 PMC9788614 36558769

[27] Esslinger JH, Smith JL. 1979. *Dipetalonema* (*Acanthocheilonema*) *didelphis* sp. n. (Nematoda: Filarioidea) from opossums, with a redescription of *D.* (*A.*) *pricei* (Vaz and Pereira 1934). Journal of Parasitology, 65, 928–933. 575550

[28] Gabrielli S, Giannelli A, Brianti E, Dantas-Torres F, Bufalini M, Fraulo M, La Torre F, Ramos RAN, Cantacessi C, Latrofa MS, Cancrini G, Otranto D. 2014. Chronic polyarthritis associated to *Cercopithifilaria bainae* infection in a dog. Veterinary Parasitology, 205, 401–404. 25037896 10.1016/j.vetpar.2014.06.027

[29] Henning TC, Orr JM, Smith JD, Arias JR, Rasgon JL, Norris DE. 2016. Discovery of filarial nematode DNA in *Amblyomma americanum* in Northern Virginia. Ticks and Tick-Borne Diseases, 7, 315–318. 26707835 10.1016/j.ttbdis.2015.11.007 PMC4876860

[30] Huson DH, Bryant D. 2006. Application of phylogenetic networks in evolutionary studies. Molecular Biology and Evolution, 23, 254–267. 16221896 10.1093/molbev/msj030

[31] Ionică AM, D’Amico G, Mitková B, Kalmár Z, Annoscia G, Otranto D, Modrý D, Mihalca AD. 2014. First report of *Cercopithifilaria* spp. in dogs from Eastern Europe with an overview of their geographic distribution in Europe. Parasitology Research, 113, 2761–2764. 24825312 10.1007/s00436-014-3931-8

[32] Ko RC. 1972. The transmission of *Ackertia marmotae* Webster, 1967 (Nematoda: Onchocercidae) of groundhogs (Marmota monax) by *Ixodes cookei*. Canadian Journal of Zoology, 50, 437–450. 5022070 10.1139/z72-062

[33] Koual R, Buysse M, Grillet J, Binetruy F, Ouass S, Sprong H, Duhayon M, Boulanger N, Jourdain F, Alafaci A, Verdon J, Verheyden H, Rispe C, Plantard O, Duron O. 2023. Phylogenetic evidence for a clade of tick-associated trypanosomes. Parasites and Vectors, 16, 3. 36604731 10.1186/s13071-022-05622-y PMC9817367

[34] De La Fuente J, Estrada-Pena A, Venzal JM, Kocan KM, Sonenshine DE. 2008. Overview: Ticks as vectors of pathogens that cause disease in humans and animals. Frontiers in Bioscience: A Journal and Virtual Library, 13, 6938–6946. 18508706 10.2741/3200

[35] Latrofa MS, Dantas-Torres F, Giannelli A, Otranto D. 2014. Molecular detection of tick-borne pathogens in *Rhipicephalus sanguineus* group ticks. Ticks and Tick-Borne Diseases, 5, 943–946. 25113982 10.1016/j.ttbdis.2014.07.014

[36] Lefoulon E, Bain O, Bourret J, Junker K, Guerrero R, Cañizales I, Kuzmin Y, Satoto TBT, Cardenas-Callirgos JM, de Souza Lima S, Raccurt C, Mutafchiev Y, Gavotte L, Martin C. 2015. Shaking the tree: Multi-locus sequence typing usurps current onchocercid (Filarial Nematode) phylogeny. PLoS Neglected Tropical Diseases, 9, e0004233. 26588229 10.1371/journal.pntd.0004233 PMC4654488

[37] Lineberry MW, Sundstrom KD, Little SE, Stayton EM, Allen KE. 2020. Detection of *Cercopithifilaria bainae* infection in shelter dogs and ticks in Oklahoma, USA. Parasites & Vectors, 13, 1–6. 32334624 10.1186/s13071-020-04089-z PMC7183667

[38] Londono I. 1976. Transmission of microfilariae and infective larvae of *Dipetalonema viteae* (Filarioidea) among vector ticks, *Ornithodoros tartakowskyi* (Argasidae), and loss of microfilariae in coxal fluid. Journal of Parasitology, 62, 786–788. 988155

[39] Namrata P, Miller JM, Shilpa M, Reddy PR, Bandoski C, Rossi MJ, Sapi E. 2014. Filarial nematode infection in *Ixodes scapularis* ticks collected from Southern Connecticut. Veterinary Sciences, 1, 5–15.

[40] Olmeda-Garcia AS, Rodriguez-Rodriguez JA. 1994. Stage-specific development of a filarial nematode (*Dipetalonema dracunculoides*) in vector ticks. Journal of Helminthology, 68, 231–235. 7829843 10.1017/s0022149x00014395

[41] Olmeda-García AS, Rodríguez-Rodríguez JA, Rojo-Vázquez FA. 1993. Experimental transmission of *Dipetalonema dracunculoides* (Cobbold 1870) by R*hipicephalus sanguineus* (Latreille 1806). Veterinary Parasitology, 47, 339–342. 8333138 10.1016/0304-4017(93)90034-k

[42] Otranto D. 2015. Diagnostic challenges and the unwritten stories of dog and cat parasites. Veterinary Parasitology, 212, 54–61. 26100153 10.1016/j.vetpar.2015.06.002

[43] Otranto D, Brianti E, Latrofa M, Annoscia G, Weigl S, Lia R, Gaglio G, Napoli E, Giannetto S, Papadopoulos E, Mir G, Dantas-Torres F, Bain O. 2012. On a *Cercopithifilaria* sp. transmitted by *Rhipicephalus sanguineus*: A neglected, but widespread filarioid of dogs. Parasites & Vectors, 5, 1–9. 22212459 10.1186/1756-3305-5-1 PMC3259067

[44] Petit G, Bain O, Carrat C, De Marval F. 1988. Développement de la Filaire *Monanema martini* dans l’épiderme des tiques Ixodidae. Annales de Parasitologie Humaine et Comparée, 63, 54–63. 3400961 10.1051/parasite/198863154

[45] Petit G, Bain O, Cassone J, Seureau C. 1988. La filaire *Cercopithifilaria roussilhoni* chez la tique vectrice. Annales de Parasitologie Humaine et Comparée, 63, 296–302. 3202588 10.1051/parasite/1988634296

[46] Ramos RAN, Giannelli A, Dantas-Torres F, Mallia E, Passantino G, Lia RP, Latrofa MS, Mutafchiev Y, Otranto D. 2013. *Cercopithifilaria rugosicauda* (Spirurida, Onchocercidae) in a roe deer and ticks from southern Italy. International Journal for Parasitology: Parasites and Wildlife, 2, 292–296. 24533349 10.1016/j.ijppaw.2013.09.009 PMC3862540

[47] Sazmand A, Bahiraei Z, Nemati F, Annoscia G, Bezerra-Santos MA, Nayebzadeh H, Salemi AM, Mousavi SM, Sadjjadi SM, Otranto D. 2022. Dermal microfilariae of dogs, jackals and cats in different regions of Iran. Parasites & Vectors, 15, 1–9. 35057824 10.1186/s13071-021-05141-2 PMC8772098

[48] Solinas C, Varcasia A, Brianti E, Giannetto S, Pipia AP, Columbano N, Tosciri G, Dantas-Torres F, Garippa G, Otranto D, Scala A. 2014. *Cercopithifilaria* spp. in dogs in Sardinia Island (Italy). Parasitology Research, 113, 675–679. 24271152 10.1007/s00436-013-3695-6

[49] Spratt DM, Haycock P. 1988. Aspects of the life history of *Cercopithifliraia johnstoni* (Nematoda: Filarioidea). International Journal for Parasitology, 18, 1087–1092. 3220649 10.1016/0020-7519(88)90079-3

[50] Tokarz R, Tagliafierro T, Ian Lipkin W, Marques AR. 2020. Characterization of a *Monanema* nematode in *Ixodes scapularis*. Parasites & Vectors, 13, 371. 32709241 10.1186/s13071-020-04228-6 PMC7379800

[51] Uni S, Bain O, Fujita H, Matsubayashi M, Fukuda M, Takaoka H. 2013. Infective larvae of *Cercopithifilaria* spp. (Nematoda: Onchocercidae) from hard ticks (Ixodidae) recovered from the Japanese serow (Bovidae). Parasite, 20, 1. 23340227 10.1051/parasite/2012001 PMC3718534

[52] Winkhardt HJ. 1980. The larval development of *Dipetalonema rugosicauda* (syn. *Wehrdikmansia rugosicauda*) in the tick *Ixodes ricinus*. II. The development of *Dipetalonema rugosicauda* in *Ixodes ricinus* and investigations about the occurrence of the microfilariae in the roe deer (*C. capreolus*). Tropenmedizin und Parasitologie, 31, 21–30. 7189613

[53] Yates JA, Lowrie RC. 1984. Development of *Yatesia hydrochoerus* (Nematoda: Filarioidea) to the infective stage in ixodid ticks. Proceedings of the Helminthological Society of Washington, 51, 187–190.

