# Supplementary material for: Multi-locus sequence analysis unveils a novel genus of filarial nematodes associated with ticks in French Guiana
Source: Parasite. 2024 Mar 15;31:14. doi: 10.1051/parasite/2024015 (PMC10941835; doi:10.1051/parasite/2024015)
Supplement: Supplementary file 1 — Table S1: List and description of primers used in this study for DLF molecular typing (adapted from Lefoulon et al., 2015). [file parasite-31-14-s1.pdf]

Table S1. List and description of primers used in this study for DLF molecular typing (adapted from Lefoulon et al., 2015).

Gene	Hypothetical product	Primers (5'-3')		Tm	Fragment size
MyoHC	Myosin heavy chain	fil_myoHCp_F1	GCATCARGAAGAAATTAATCG	52°C	Nested PCR assay:
		fil_myoHCp_R1	GCTTCAATTTCYTCCTCCAT		1st round PCR: fil_myoHCp_F1 / fil_myoHCp_R1: 1060 bp
		fil_myoHCpN_F2	GAYGATCTTGAGGACAAYYT		2nd round PCR: fil_myoHCpN_F2/fil_myoHCpN_R2: 785 bp
		fil_myoHCpN_R2	TCTTCAATYTGTTTBCCRAG		
hsp70	Chaperone protein (heat shock protein)	FHsp70_dF3	TCRGATTTCTTYTCTGGYA	52°C	Semi- Nested PCR assay:
		FHsp70_dR1	GTYTGYTTCATATTGAAYGC		1st round PCR: FHsp70_dF3/FHsp70_dR1: 710bp
		FHsp70_dF1	CAGCTATYCTYTCTGGTGAT		2nd round PCR: FHsp70_dF1/ FHsp70_dR1: 610bp
rbp1	RNA polymerase II large subunit	RNApolyLSp_dF1	ACTGCAAAYACWGCWATTTA	52°C	Nested PCR assay:
		RNApolyLSp_dR1	ACRTGATTCATTTCRCGTTC		1st round PCR :RNApolyLSp_dF1/RNApolyLSp_dR1: 640bp
		RNApolyLSp_dF1b	TAATTGAAGAAGATCAGGAGTG		2nd round PCR: RNApolyLSp_dF1b/RNApolyLSp_dR1b: 544bp
		RNApolyLSp_dR1b	CGAATATTTCACAGATATCGT		
12S rRNA	SSU rRNA of mitochondrial ribosome	12SdegF2	ATTACYTATTYTTAGTTTA	50°C	Nested PCR assay:
		12SnemR2	CTACCATACTACAACTTACGC		1st round PCR: 12SdegF2/12SnemR2: 600pb
		12SF	GTTCCAGAATAATCGGCTA		2nd round PCR: 12SF/12SdegR: 450bp
		12SdegR	ATTGACGGATGRTTTGTACC		
28S rRNA	LSU rRNA of eukaryotic cytoplasmic ribosome	F28SF1	CCTCAACTCAGTCGTGATTACC	55°C	Semi-nested PCR assay:
		F28SR2	CTCTGGCTTCATCCTGCTCA		1st round PCR: F28SF1/F28SR2: 1150bp
		F28SintdR1	TCTTYACTTTCATTAYGCTT		2nd round PCR: F28SF1/F28SintdR1: 970bp
18S rRNA	SSU rRNA of eukaryotic cytoplasmic ribosome	F18ScF1	ACCGCCCTAGTTCTGACCGTAAA	52°C	Simple PCR assay:
		F18ScR1	GGTTCAAGCCACTGCGATTAAAGC		PCR: F18ScF1/F18ScR1: 740bp
