# Supplementary material for: Multi-locus sequence analysis unveils a novel genus of filarial nematodes associated with ticks in French Guiana
Source: Parasite. 2024 Mar 15;31:14. doi: 10.1051/parasite/2024015 (PMC10941835; doi:10.1051/parasite/2024015)
Supplement: Supplementary file 2 — Table S2: List of GenBank accession numbers for MyoHC, hsp70, rbp, 12S rRNA, 28S rRNA, and 18S rRNA gene sequences used in phylogenetic analyses. [file parasite-31-14-s2.pdf]

Table S2. List of GenBank accession numbers for *MyoHC*, *hsp70*, *rbp*, 12S rRNA, 28S rRNA, and 18S rRNA gene sequences used in phylogenetic analyses.

Espèce	Sample ID	coxI	MyoHC	rbp1	hsp70	12S rRNA	28S rRNA	18S rRNA
Acanthocheilonema odendhali	401YU	KP760168	KP760212	KP760263	KP760410	KP760314	KP760358	KP760116
Acanthocheilonema vitaea	7YT	KP760169	KP760213	KP760264	KP760411	KP760315	KP760359	KP760117
Cercopithifilaria bainae	9YT3	KP760175	KP760219	KP760271	KP760417	KP760321	KP760365	KP760123
Cercopithifilaria rugosicauda	350YU	KC610815	KP760220	KP760272	KP760418	KC610812	KP760366	KP760124
Monanema martini	31NC	KP760196	KP760244	KP760295	KP760442	FR827911	KP760391	KP760148
Yatesia hydrochoerus	52YT	KP760211	KP760261	KP760312	KP760459	KP760356	KP760409	KP760166
Cruorifilaria tuberocauda	55YT	KP760176	KP760221	KP760273	KP760419	KP760322	KP760367	KP760125
Litomosoides brasiliensis	PF35	KP760190	KP760235	KP760286	KP760433	KP760335	KP760381	KP760139
Litomosoides brasiliensis	PF37	KP760191	KP760236	KP760287	KP760434	KP760336	KP760382	KP760140
Litomosoides hamletti	PF36	KP760192	KP760237	KP760288	KP760435	KP760337	KP760383	KP760141
Litomosoides solarii	213YU	KP760193	KP760238	KP760289	KP760436	KP760338	KP760385	KP760142
Dipetalonema caudispina	362YU	KP760177	KP760222	KP760274	KP760420	KP760323	KP760368	KP760126
Dipetalonema caudispina	64YT	KP760178	KP760223	KP760275	KP760421	KP760324	KP760369	KP760127
Dipetalonema gracile	124CV	KP760179	KP760224	KP760276	KP760422	KP760325	KP760370	KP760128
Dipetalonema gracile	215YU	KP760180	KP760225	KP760277	KP760423	KP760326	KP760371	KP760129
Dipetalonema gracile	63YT	KP760181	KP760226	KP760278	KP760424	KP760327	KP760372	KP760130
Dipetalonema graciliformis	220YU	KP760182	KP760227	KP760279	KP760425	KP760328	KP760373	KP760131
Dipetalonema robini	217YU	KP760183	KP760228	KP760280	KP760426	KP760329	KP760374	KP760132
Brugia malayi	8YT	KP760171	KP760216	KP760267	KP760414	KP760317	KP760362	KP760120
Brugia pahangi	46YT	KP760172	KP760217	KP760268	KP760415	KP760318	KP760363	KP760121
Brugia timori	6YT	KP760173	KP760218	KP760269	KP760416	KP760319	KP760364	KP760122
Loa loa	80YT	KP760194	KP760239	KP760290	KP760437	KP760339	KP760386	KP760143
Foleyella candezei	68CE	KP760187	KP760232	KP760283	KP760430	FR827906	KP760378	KP760136
Pelecitus fulicaeatrae	49YT	KP760206	KP760256	KP760308	KP760454	KP760352	KP760404	KP760161
Breinlia jittapalapongi	78YT	KP760170	KP760215	KP760266	KP760413	KP760316	KP760361	KP760119
Aproctella alessandroi	117YU	FR823335	KP760214	KP760265	KP760412	FR827905	KP760360	KP760118
Rumenfilaria andersoni	94YU	JQ888273	KP760258	KP760309	KP760456	JQ888291	KP760406	KP760163
Madathamugadia heipei	81YU	JQ888270	KP760242	KP760293	KP760440	JQ888289	KP760389	KP760146
Mansonella ozzardi	77YT	KP760195	KP760243	KP760294	KP760441	KP760340	KP760390	KP760147
Mansonella perforata	216JW	AM749265	KP760241	KP760292	KP760439	AM779802	KP760388	KP760145
Onchocerca armilatta	54FKa1	KP760200	KP760248	KP760300	KP760446	KP760345	KP760396	KP760153
Onchocerca dewittei japonica	OB9	KP760203	KP760249	KP760301	KP760447	KP760349	KP760397	KP760154
Onchocerca eberhardi	S63-5	AM749268	KP760250	KP760302	KP760448	KP760346	KP760398	KP760155
Onchocerca gutturosa	54FKg1	KP760201	KP760251	KP760303	KP760449	KP760347	KP760399	KP760156
Onchocerca ochengi	54FKo1	KP760202	KP760252	KP760304	KP760450	KP760348	KP760400	KP760157
Onchocerca skrjabini	S63-6	AM749269	KP760253	KP760305	KP760451	AM779806	KP760401	KP760158
Dirofilaria immitis	79YT	KP760184	KP760229	KP760281	KP760427	KP760330	KP760375	KP760133
Dirofilaria repens	297YU	KP760185	KP760230	KP760282	KP760428	KP760331	KP760376	KP760134
Setaria labiatopapillosa	413YU	KP760208	KP760259	KP760310	KP760457	KP760354	KP760407	KP760164
Setaria tundra	71YT	KP760209	KP760260	KP760311	KP760458	KP760355	KP760408	KP760165
Icosiella neglecta	44YT	KP760188	KP760233	KP760284	KP760431	KP760333	KP760379	KP760137
Oswaldofilaria chabaudi	191YU	KP760204	KP760254	KP760306	KP760452	KP760350	KP760402	KP760159
Oswaldofilaria petersi	PF34	KP760205	KP760255	KP760307	KP760453	KP760351	KP760403	KP760160
Wuchereria bancrofti	17/3	SSBO01000000	SSBO01000000	SSBO01000000	SSBO01000000	SSBO01000000	SSBO01000000	SSBO01000000
Ascaris suum	RED_2019	JACCHR010000000	JACCHR010000000	JACCHR010000000	JACCHR010000000	JACCHR010000000	JACCHR010000000	JACCHR010000000
